# Supplementary material for: Cervical cancer: Riverside women’s knowledge in the Brazilian Amazon about preventive measures
Source: PLoS One. 2026 May 20;21(5):e0347609. doi: 10.1371/journal.pone.0347609 (PMC13189305; doi:10.1371/journal.pone.0347609)
Supplement: S1 File — (PDF) [file pone.0347609.s001.pdf]

UNIVERSITY OF THE STATE OF PARÁ
CENTER FOR BIOLOGICAL AND HEALTH SCIENCES
GRADUATE COURSE IN NURSING

APPENDIX A - DATA COLLECTION INSTRUMENT

Cervical cancer: knowledge of riverine women about preventive measures.

PART I – PROFILE:

Identification Code: _______ Date:____/____/_____

Age: ______ years Race/Ethnicity: ___________

Education level: ______________ Religion: ______________

Sex: ______________ Marital Status: ______________

Occupation: ______________ Do you have children? ( ) yes ( ) no

If yes, how many?

Household income (in minimum wages):

Are you currently sexually active? ( ) yes ( ) no

PART II – ABOUT THE RESEARCH TOPIC:

1. What do you know about cervical cancer?

   - In your opinion, what is it?
   - How do you think this cancer develops?
   - What do you think can cause cervical cancer?
   - Do you think there are factors (such as age, race/ethnicity, etc.) that may influence the development of cervical cancer, or can anyone develop it?
   - What tests do you know that can detect cervical cancer?

2. How do you think you can protect yourself against this cancer?

   - What actions do you think can be taken to prevent cervical cancer?
   - What guidance have you received on how to prevent cervical cancer?
   - Who has talked to you about these preventive measures? In what settings have these discussions taken place?
   - How do you think healthcare professionals contribute to prevention?

- Do you think that condom use can help prevent cervical cancer in any way? Why?
- What do you currently do to help protect yourself against cervical cancer?

3. Are you familiar with the Human Papillomavirus (HPV)?

- What do you know about it?
- What can it cause?
- How can it be transmitted?
- What actions do you think reduce the risk of infection?

4. What do you observe during healthcare consultations related to cervical cancer, whether in prevention or guidance?

5. If you have had any experience with cancer in your community, how has it affected your current way of life?

6. Do you use any contraceptive method? If so, which ones?
