# Supplementary material for: Cervical cancer: Riverside women’s knowledge in the Brazilian Amazon about preventive measures
Source: PLoS One. 2026 May 20;21(5):e0347609. doi: 10.1371/journal.pone.0347609 (PMC13189305; doi:10.1371/journal.pone.0347609)
Supplement: S2 File — (PDF) [file pone.0347609.s002.pdf]

UNIVERSIDADE DO ESTADO DO PARÁ
CENTRO DE CIÊNCIAS BIOLÓGICAS E DA SAÚDE
CURSO DE GRADUAÇÃO EM ENFERMAGEM

APÊNDICE A - INSTRUMENTO PARA A COLETA DE DADOS

Câncer de colo do útero: conhecimento de mulheres ribeirinhas sobre medidas preventivas

PARTE I – PERFIL:

Código de Identificação: _______ Data:____/____/_____

Idade: ______ anos Raça/Cor: ___________

Escolaridade: ______________ Religião: ______________

Sexo: ______________ Estado civil: ______________

Ocupação: ______________ Tem filhos? ( ) sim ( ) não

Se sim, Quantos?

Renda familiar (em salários mínimos):

Tem vida sexual ativa? ( ) sim ( ) não

PARTE II – SOBRE O OBJETO DE ESTUDO:

1. O que você sabe sobre o câncer de colo de útero?

- Na sua opinião, o que é?
- Como você acha que esse câncer se desenvolve?
- O que você acha que pode causar o câncer de colo de útero?
- Você acha que existe algum tipo de influência (idade, raça/cor…) para que a possa ter o câncer de colo uterino ou qualquer pessoa pode ter?
- Que exames você conhece que podem fazer a pessoa descobrir que tem câncer de colo de útero?

2. Como você acha que pode se proteger contra esse câncer?

- Que ações você acha que pode fazer para prevenir o câncer de colo de útero?
- Que orientações você já recebeu de como prevenir o câncer de colo de útero?
- Quais pessoas já falaram com você sobre essas formas de prevenção? Em que ambientes isso já foi conversado?

- Como você acha que os profissionais de saúde contribuem com as medidas de prevenção?

- Você acha que o uso de preservativos pode ajudar a evitar de alguma forma o aparecimento do câncer? Por quê?

- O que você faz hoje em dia que contribui para se proteger contra o câncer de colo de útero?

3. Você conhece o Papiloma Vírus Humano (HPV)?

- O que você sabe sobre ele?
- O que ele pode causar?
- Como pode pegar?
- Que ações você acha que diminuem o risco de pegar esse vírus?

4. O que você vê durante o atendimento de saúde que é voltado para o câncer de colo de útero, seja na prevenção ou nas orientações?

5. Caso você tenha tido alguma experiência com o câncer no meio em que vive, como isso impactou no modo de vida hoje em dia?

6. Você utiliza algum método contraceptivo? Se sim, quais?
