# Supplementary material for: Cervical cancer: Riverside women’s knowledge in the Brazilian Amazon about preventive measures
Source: PLoS One. 2026 May 20;21(5):e0347609. doi: 10.1371/journal.pone.0347609 (PMC13189305; doi:10.1371/journal.pone.0347609)
Supplement: Attachment — Submitted filename: Response to Reviewers.pdf [file pone.0347609.s003.pdf]

LETTER TO REVIEWERS

We thank the reviewers for their relevant contributions and for their kindness in evaluating the manuscript. When reviewing the manuscript, we took into account their suggestions/recommendations, always aiming to improve the quality of the text. In the **manuscript (revised version with change control),** the adjustments are highlighted with a **green bar**. As instructed by the journal, we would like to point out that, in addition to this version with change control, we have also attached a version without markings, the content of which is similar to that of the version with markings.

We are available for any further assistance. Below, we present the reviewers' comments and suggestions/recommendations, with the expressions **"1) ACADEMIC EDITOR'S OPINION"** and **"2) REVIEWER'S OPINION"**, as well as our respective comments.

1) ACADEMIC EDITOR'S OPINION:

Dear author,

- After careful analysis, I feel that the manuscript does not fully meet the PLoS ONE publication criteria, as it currently stands. (**AUTHORS' COMMENT:** We are aware! We have made the necessary efforts to improve the text and increase its potential to contribute to the advancement of knowledge, according to the suggestions/recommendations of the reviewers. Therefore, we understand that the text, as it stands at this time, is capable of continuing the evaluation and editorial processes, with a view to possibly obtaining acceptance for publication).

- The manuscript explores a very important topic, but there are weaknesses in the results – they are not clear. (**AUTHORS' COMMENT:** Before being submitted to PLoS ONE, the text underwent rigorous language review, which was signed by the technical manager of *LSB Traduções* [*Cadastro Nacional de Pessoa Jurídica* – CNPJ: 28.340.353/0001-50], a reputable company that provides text review and translation services in Brazil. As proof of its respectability, this company was accredited by several qualified scientific journals in the field of nursing in the country, such as *Acta Paulista de Enfermagem* [APE – ISSN: 1982-0194], *Revista Brasileira de Enfermagem* [REBEn – ISSN: 1984-0446) and *Revista da Escola de Enfermagem da USP* [REEUSP – ISSN: 1980-220X]. Therefore, we respectfully understand that the Results meet the textual clarity required to be understood, which is why the *Ad Hoc* Reviewer, who evaluated this manuscript, did not make any comments or suggestions/recommendations that would indicate the lack of clarity in this section).

2) REVIEWER'S OPINION:

TITLE: Cervical cancer: Riverside women's knowledge about preventive measures.

- Since this is a very specific population (riverside women in the Brazilian Amazon), it would be interesting to highlight the nationality of this study in the title, since "Riverside" is a very generic term. Several articles in the journal PLoS ONE include the nationality of the study in the title, especially when it is a description of very specific characteristics of a population, with no intention of extrapolating the data, as is the case with this study.

Suggestion: "Cervical cancer: Amazonian riverside women's knowledge about preventive measures" or "Cervical cancer: Brazilian riverside women's knowledge about preventive measures". Readers more familiar with the topic will know that the Brazilian Amazon region is a "hot spot" for cervical cancer, helping the reader to know whether or not this manuscript is of interest to them. (**AUTHORS' COMMENT:** Met! We have adjusted the title to read: "Cervical cancer: Riverside women's knowledge in the Brazilian Amazon about preventive measures").

ABSTRACT:
- Objectives, Methodology, and Results: Adequate. (**AUTHORS' COMMENT:** Aware!).

- Conclusion: Wouldn't it be better to mention some of the "gaps" in the knowledge found? Wouldn't it be better to put these gaps in the results? (**AUTHORS' COMMENT:** Met! We have adjusted the Results and Conclusion of the Abstract, highlighting gaps specifically in the Results).

INTRODUCTION:
- The first two paragraphs provide information on cervical carcinogenesis, with an emphasis on the molecular role of HPV. This could be removed, as it is known information that is not relevant to this manuscript (only mentioning the role of HPV, without emphasis on molecular mechanisms). In this way, the authors could add information on studies that seek to improve screening strategies, corroborating the statistical data that the authors presented, which place the Brazilian Amazon region as a target for a closer look at this disease. (**AUTHORS' COMMENT:** Granted!).

- Therefore, for contextualization purposes, I suggest that quantitative studies on the prevalence of HPV infection in riverside and indigenous women, innovative methods of self-collection and molecular diagnosis of HPV for women living in isolated regions (riverside and indigenous) in Amazonas, Pará, Roraima be added, as we already have articles published on this, including in the journal PLoS ONE. These studies provide a wealth of information on socioeconomic and clinical-behavioral characteristics related to HPV and other sexually transmitted pathogens that are quite comparable to the results of this study and, together, provide a more complete view of the vulnerability of riverine women. (**AUTHORS' COMMENT:** Granted! In the Introduction, in general, we have removed information on the molecular mechanisms of HIV infection and incorporated studies that address the evidence and information requested by the Reviewer. Thus, the content of this section has been expanded, aiming to better direct you to the pertinent aspects of the topic).

METHODOLOGY:
- Adequate. (**AUTHORS' COMMENT:** Aware!).

RESULTS:
- The descriptive sociodemographic and clinical results would be better understood in a table (first 3 paragraphs). These data are very important, as they characterize the population under study. Although the authors described certain aspects of the Cotijuba community in the Methodology, it is these sociodemographic data that confirm the vulnerability of these women. Therefore, it is necessary to facilitate the reader's access to these data so that they can better follow the Discussion. (**AUTHORS' COMMENT:** Received! At the beginning of the Results, we created a table to detail the variables of the sociodemographic and clinical profile of the participants. Thus, in the first two paragraphs of this section, we highlight the predominant information, indicating that readers should look at the table to access the details).

DISCUSSION:
- Adequate. (**AUTHORS' COMMENT:** Aware!).
